# Vision-Less Sensing for Autonomous Micro-Drones †

**DOI:** 10.3390/s21165293

**Published:** 2021-08-05

**Authors:** Simon Pikalov, Elisha Azaria, Shaya Sonnenberg, Boaz Ben-Moshe, Amos Azaria

**Affiliations:** Computer Science Department, Ariel University, Ariel 40700, Israel; simon96pikalov@gmail.com (S.P.); elishadar@gmail.com (E.A.); sonnenberg16@gmail.com (S.S.); amos.azaria@ariel.ac.il (A.A.)

**Keywords:** autonomous micro-drones, sensor fusion, indoor mapping, bio-inspired micro-robotics

## Abstract

This work presents a concept of intelligent vision-less micro-drones, which are motivated by flying animals such as insects, birds, and bats. The presented micro-drone (named BAT: Blind Autonomous Tiny-drone) can perform bio-inspired complex tasks without the use of cameras. The BAT uses LIDARs and self-emitted optical-flow in order to perform obstacle avoiding and maze-solving. The controlling algorithms were implemented on an onboard micro-controller, allowing the BAT to be fully autonomous. We further present a method for using the information collected by the drone to generate a detailed mapping of the environment. A complete model of the BAT was implemented and tested using several scenarios both in simulation and field experiments, in which it was able to explore and map complex building autonomously even in total darkness.

## 1. Introduction

Autonomous robots are able to perform complex tasks, such as precise welding on a busy assembly line or performing daily cleaning tasks in a room. Yet, general purpose robotics is still in an early stage of development. For example, commercial drones have high-resolution cameras and sophisticated navigation sensors, yet, they are unable to land safely on trees or build a nest. Tasks that are commonly performed by small birds. Moreover, consider the case of beehives—with relatively low visual capabilities—the bees are able to conduct complex visual navigation, and perform hundreds of landings daily in dynamic wind conditions while having relatively low resolution visual sensing capabilities.

**Figure 1:**
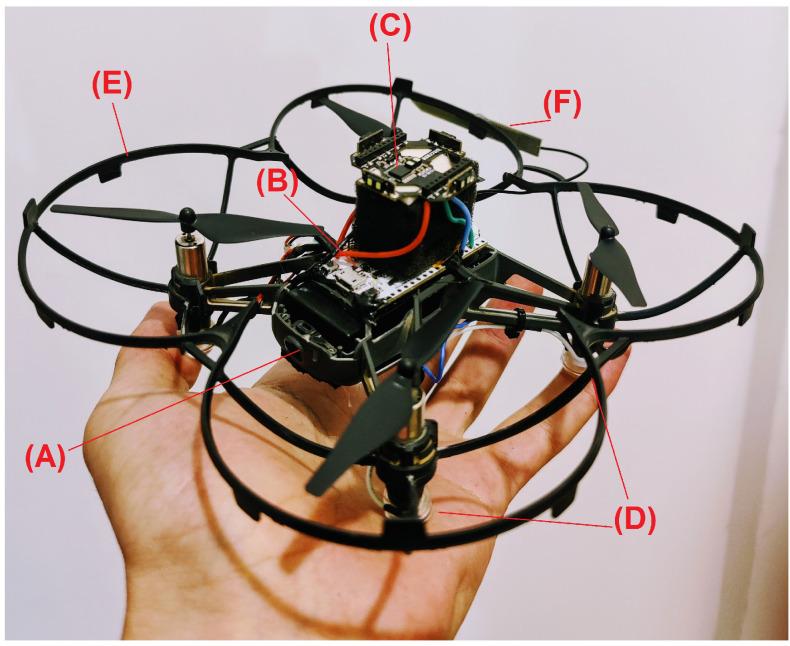
Our Blind Autonomous Tiny-drone (BAT), which is based on the Tello commercial tiny-drone (A). BAT is equipped with a multi ranger LIDAR array (front, left, right, up, and back) marked by (C), and it uses a WiFi based microcontroller (B) to control the Tello as if it was a remote control. Prop guards are used to protect BAT (E), 4x down facing LEDs are used for low light conditions (D), a small LoRa (UHF) antenna (F) is used to transmit mapping data to the mapping station.

In this paper, we focus on bio-inspired micro-drones with a weight of 80–100 g and a size of a little bird (see Figure 1). Micro-drones cause less commotion, are more flexible, consume less power, are less likely to break and cause damage if crashing, can be deployed in swarms, can operate in small passages, and are usually cheaper than large drones. The main goal of this work is to design the structure and the controlling algorithms that will allow a tiny-drone to explore and map an unknown indoor environment.

### 1.1. Related Works

The research field of bio-inspired drones has attracted researches from a wide range of backgrounds, see in [1,2] for a recent review paper regarding such applications and challenges. Motivated by the abilities of flying animals, many researchers have tried to investigate bio-mechanisms for navigation and flying, yet it seems that we are still far from being able to fully understand those methods [3]. Optical flow, which is the ability to use visual sensors in order to model an ego-movement of a robot with respect to a seen in time, is an important capability for autonomous flying drones [4]; however, up to the last decade, systems with optical flow support were relatively rare and complicated (see in [5]). Yet, recently, optical navigation capabilities became a common Commercial Off-The-Shelf (COTS) sensors, and even toy-graded drones often use optical tracking to allow the drone to maintain position. Modern flight controllers for drones, such as Pixhawk [6], support several inertial and navigation sensors (e.g., MEMS-Gyro, accelerator, magnetometer, barometer, and GNSS receiver). When fused together, these sensors allow a relatively robust navigation in outdoor conditions. In indoor flights, GNSS navigation is mostly insufficient; in such cases, cameras and range sensors are commonly used for visual navigation and obstacle detecting and avoiding [7,8]. The vision of having a sustainable swarm of autonomous aerial vehicles [9] has attracted researcheres from both academy and industry [10,11,12,13]. Yet, even the concept of a single autonomous drone still encapsulates a wide range of challenges [14,15]. Recent improvement in hardware and software for edge deep learning platforms [16,17] allows micro-drones to use visual sensors for obstacle avoidance and navigation [18]. In this paper, we present a vision-less alternative approach; we conjecture that in many real-world natural cases, the suggested framework performs better than vision-based solutions. There have been several previous attempts to deploy autonomous drones for mapping in-door environments, most of which require vision sensors. Dowling et al. [19] presented a method for mapping in-door environments using a drone. While their results seem promising, their approach uses the Erle-copter drone, which is relatively large (at least 10 times larger than BAT). In addition, their approach requires substantial computing power using a Raspberry Pi 3b. Similarly, Zhang et al. [20] proposed a method for 3D cave mapping for archaeology applications using a drone. Their proposed drone is intended for use with human support for controlling it, and thus cannot be seen as a fully autonomous drone. While the exact proposed drone type is not presented in the paper, the sensors and computer power described mitigate the possibility for using a micro-done.

Li et al. [21] propose a method for using a drone to map mine environments. They show the efficiency of their method by running experiments both in simulation and in the real world with their developed drone. They propose the use of the DJI Matrice 100 drone, which weighs over 3kg and has a clearance of over one meter. As stated, all these attempts require large drones, and thus cannot benefit from all of the advantages of micro-drones.

### 1.2. Motivation

Motivated by bio-inspired robotics, this paper considers challenges related to sustainable robotics [22]. In particular to design and construct an aerial robot that can perform some kind of robotic-life-cycle rather than dedicated predefined tasks. The micro-drone sustainability algorithm will provide it with the basic abilities needed to survive in the environment, while performing the predefined backmapping task (e.g., sensing and searching). The scope of sustainable robotics research is wide and involves multi-discipline fields of research (Robotics, Machine Learning, Multi-Agent Systems, and Human–Machine Interaction). The general use case of a swarm of autonomous micro-drones is not well defined but a general purpose mission. Therefore, in order to accomplish such vision, we start with defining the following individual (bio-inspired) capabilities for a single drone.

- Obstacle detection: this property is required for detecting hazards while flying and acting accordingly.
- Path planning: smart planning of the flying path is crucial for the drone resource saving and mission efficiency.
- Mapping: allowing the micro-drone to map and learn its environment, in a way that the next mission can benefit from information the drone has acquired in the last one; moreover, this info can be shared between drones.
- Communicate with others: allowing drones to share information and perform calibrated missions.

This research focuses on the notion of sustainable robotics for a single micro drone that is both autonomous and does not need vision for navigation.

### 1.3. Our Contribution

In this paper, we present a new concept of vision-less bio-inspired micro-drone, which is able to perform complex missions such as obstacle avoidance, navigating, and mapping without the need of a camera. By using micro single-point range sensors such as optical-flow and time-of-flight (ToF) ranging sensors, we were able to design autonomous controlling algorithms applicable for on-board flight controllers with limited computing power (microcontroller). We constructed a Blind Autonomous micro-drone (BAT). BAT is a standalone autonomous aerial vehicle, in which all computation is performed on-board. BAT sends the information to a mapping station in order to construct a graphic representation and a mapping. To the best of our knowledge, this is the first work that presents a fully autonomous vision-less micro-drone capable of mapping in indoor settings.

The remainder of the paper is constructed as follows. Section 2 covers BAT’s platform in terms of standard sensors and additional hardware required for autonomous flying. Next, in Section 3 we present the main autonomous control algorithm, allowing our drone to perform obstacle avoidance exploration of a complex regions. Then, in Section 4 we present the mapping capabilities of BAT. Section 5 describes our experimental evaluation in both simulation and field experiments. Finally, in Section 6 we conclude the research and discuss several related future problems.

## 2. BAT Modeling Hardware

This section presents the basic hardware and software functionality of BAT. We start by presenting the COTS micro-drone (Tello), which we use. Then, we present the additional sensors and microcontrollers we have added to the drone in order to make it autonomous. Finally, we describe the configuration of the (optional) mapping station, which is used for real-time visualization and mapping.

### 2.1. Tello Drone

The Tello drone is manufactured by Ryze Tech and powered by DJI. It has a weight of 80 g, dimensions of 98 mm × 92.5 mm × 41 mm, and 3 inch propellers. The drone is equipped with a suite of sensors on-board including an optical flow sensor, accelerometer, gyro, barometer, and a Time of Flight (ToF) range sensor. Interfacing with the drone can be done via WiFi using the companion SDK (provided by DJI). The Optical Flow sensor, located at the bottom of the drone facing the ground, can calculate the change in the *X* and *Z* coordinates over time. This sensor is basically a low-resolution image sensor (camera), taking images every time interval, comparing consecutive frames and deriving the change in each coordinate. The Optical Flow sensor assists the drone with hovering over a given location without drift, it is also used to calculate the drone’s relative velocity. The optical flow sensor is only used internally by the drone in order to compute the velocity, and cannot be accessed directly. The ToF range sensor, located at the bottom of the drone facing the ground, is used to determine the relative height of the drone from an object below it, which is essential for climbing staircases and avoiding obstacles from below. Using the barometer the drone can detect a relative altitude (height) with respect to the takeoff point.

### 2.2. Companion Hardware

In addition to the hardware already available in the Tello drone, we added three components: a microcontroller, a multi-ranger LiDAR deck, and LEDs.

#### 2.2.1. MicroController

To command BAT autonomously and to process the various sensor data, an ESP32 based microcontroller was used; we have mainly focused on the WiFi LoRa 32 (V2) by HELTEC. The small-factor microcontroller weighs about 5 g and has an ESP32 (dual-core 32-bit MCU + ULP core) Microprocessor, which includes WiFi, Bluetooth, and a LoRa node chip (SX1278) with its external antenna.

#### 2.2.2. Multi-Ranger LiDAR Deck

The sensor array used in this project is a COTS PCB (made by Bitcraze) that has 5× VL53L1X LiDAR-based range sensors. The sensors are facing front, back, left, right, and up. Combining these sensors with the ToF sensor facing down on the drone, we can achieve a 360-degree range coverage. The VL53L1X sensors are a ToF laser-ranging sensor. With low power composition and a tiny package, the sensors are capable of accurately measuring distance up to 4 m at 25 Hz. See Appendix A for further details.

#### 2.2.3. LEDs

The optical flow present in the Tello drone requires at least 100 lumens per square meter (lux) in order to function properly; this may be challenging in a dark environment. The optical flow is not only used for obtaining measurements required by the mapping, but a lack in lighting conditions may cause the drone to drift even when not receiving any commands from the microcontroller. Furthermore, the Tello drone does not accept any commands from the microcontroller in poor lighting conditions. Therefore, we added 4x down-facing LEDs to BAT’s legs in order to provide additional lighting; in practice, the LEDs provide enough light even in an absolutely dark environment.

### 2.3. Communication

**Figure 2:**
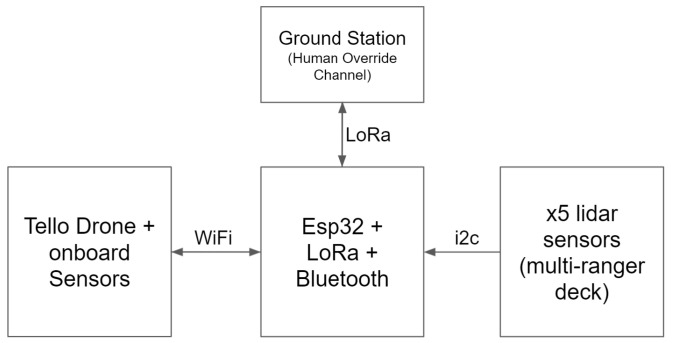
The communication diagram of BAT. BAT has a companion computer based on an ESP32 microcontroller which supports WiFi, Bluetooth, and LoRa. The WiFi is controlling the (original) Tello as if it was a remote control, the communication with the mapping station is performed via Long Range (LoRa), while the Bluetooth modem is reserved for short range debugging and in the future can be served as a base for drone to drone (mesh) communication.

Several different communication protocols were used in this project. The drone and ESP32 communicate over WiFi utilizing two UDP ports, one for commanding the drone using both RC commands (throttle, roll, pitch, and yaw) and higher level commands (i.e., “takeoff”, “land”, etc.), and the second port is for getting the information from the drone’s on-board sensors back from the drone. The multi-ranger deck is connected to the $I^{2}C$ bus. After assigning each sensor its unique $I^{2}C$ address, all of the sensors can be addressed independently. LoRa communication is used to send the data back to the mapping station, human intervention if needed is also accomplished via LoRa communication. Bluetooth is also used to send the data back to the mapping station, this link is less reliable and is used mainly when BAT is close to the mapping station. See Figure 2 for a communication diagram.

### 2.4. Mapping Station

The mapping station is used to create a live map from BAT’s output data All the relevant data are transmitted from the drone back to the mapping station, the data are used for creating a live 3d map of the environment. The live map updates as soon as new data arrives. Alternatively, instead of transmitting to the mapping station, one can store the data on-board using an SD card adapter, and perform the mapping offline. In addition, the mapping station may be equipped with a component that allows controlling the drone in case of an emergency, or sending high level commands such as returning to takeoff point. This is achieved using an esp32 based microcontroller to establish a 2-way LoRa communication between the drone and the mapping station.

### 2.5. Controlling API for Drones

Drones are commonly controlled using four channels API: Throttle, Yaw, Pitch, and Roll. The control channels are defined with respect to the drone local coordinate system (*X*-right, *Y*-up, *Z*-front). The Throttle channel controls the overall force (power) of the drone’s motors (*Y*-axis acceleration). The Yaw channel controls the angular velocity of the drone with respect to the *Y*-axis (Gravitation). The Pitch channel controls the angle of the front of the drone with respect to the horizon (i.e., *Z*-axis acceleration). The Roll channel controls the angle of the right side of the drone (*X*-axis acceleration).

## 3. Controlling Algorithm

Recall that BAT’s goal is to explore and map an unknown indoor environment. Therefore, its goal is to maximize the newly visited regions and not revisiting known areas or traveling in an endless loop, while performing a safe flight and avoiding obstacles. To that end, BAT’s control algorithm is based on the concept of the wall follower algorithm, which follows the right wall [23]. Clearly, the algorithm can be mirrored by following the left wall, which can be useful if BAT wishes to return to the takeoff point. BAT has the following discrete states:

- Ground: BAT is on the ground. This is the initial state.
- Takeoff: BAT starts flying upwards and gets to a predefined altitude (e.g., 1 m).
- Control: The main control loop.
  Rotate C.C.W.: BAT slightly rotates counterclockwise (to align with the right wall).
  Emergency: BAT brakes to avoid crashing.
  Tunnel: BAT centers in between the left and right walls while maintaining the desired speed.
  Turn C.W.: BAT turns 90 degrees clockwise (to find the right wall).
  Fly Forward: BAT flies forward while making minor adjustments to maintain predefined bounds, i.e., its distance from the right and the desired speed.

**Figure 3:**
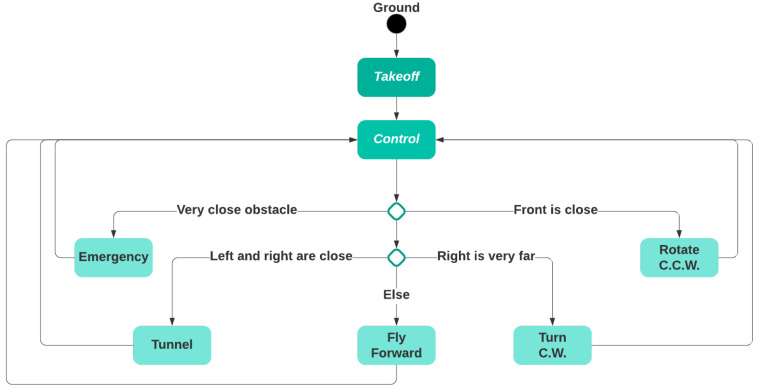
BAT’s controlling state machine.

The high-level control algorithm role is to select the next state from the five possible states (Rotate C.C.W., Emergency, Tunnel, Turn C.W., Fly Forward), each time the control state is reached (see Figure 3).

BAT’s desired value in the Roll, Pitch, and Throttle is determined based on the range from the corresponding direction using a proportional-integral-derivative (PID) controller for each controlling channel [24]. A PID controller is not used for the Yaw because it is controlled by the logic of the state machine to enable right wall navigation. When there are no obstacles, BAT accelerates until it reaches the predefined maximal speed and height.

Before we provide the PID controller formula, we introduce the following notation. *g* represents the desired goal value. e[t] represents the current error, i.e., the difference between *g* and the current measurement. u[t] denotes the weighted combined value computed by the controller at time *t*. $K_{p}$, $K_{i}$, and $K_{d}$ are manually defined constants that represent the proportional, integral, and derivative gain, i.e., the weight given to the current, past, and predicted future error, respectively.

Finally, the PID is based on the following formula:

(1) $$\begin{array}{c} u\left(t\right)=K_{p}\cdot e\left(t\right)+K_{i}\int e\left(t\right)\Delta t+K_{d}\cdot \frac{\Delta e}{\Delta t} \end{array}$$

Note that in order to allow a robust and reliable controller, it is a recommended practice to have a constraint-range for the controller output.

BAT’s control loop logic is described in Algorithm 1. The constants as implemented in BAT controllers can be found in the Appendix A.
**Algorithm 1:** BAT’s control loop logic
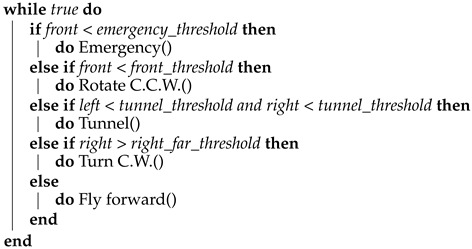


## 4. Mapping

**Figure 4:**
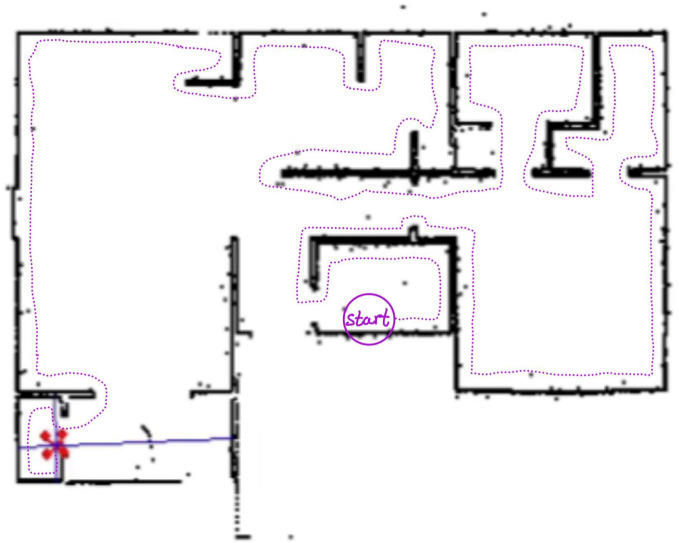
A 2D mapping of a building as computed in the simulator. BAT is marked in red, its path is marked by a dotted magenta trace, its current ranging is marked in blue rays, and the building 2D map is marked by black dots and lines.

**Figure 5:**
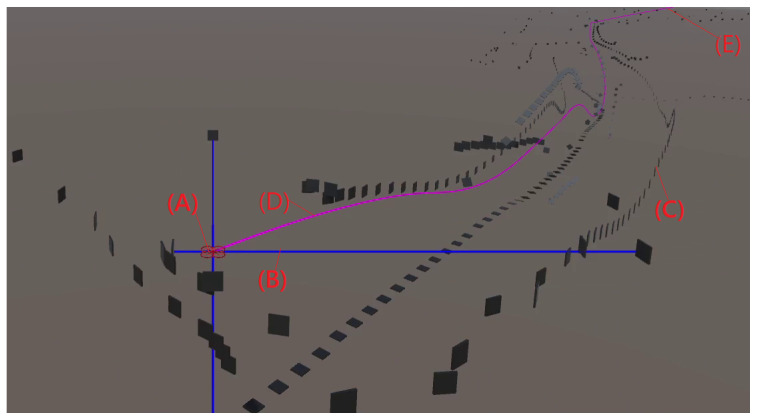
A 3D mapping of a building as transmitted by an actual BAT to the mapping station. BAT (A) is marked in red, its current ranging (B) is marked in blue rays, the path (D) of BAT is marked in magenta, and its starting point is marked as (E). The building 3D point-clouds (C) is marked by black tiles.

The main goal of BAT is to provide a mapping of an indoor environment. The mapping is performed using a coordinate system that is relative to BAT’s starting point and orientation; we denote this coordinate system as the global one. The mapping station performs the mapping based on the information received from BAT. The mapping station computes a geometrical model of the environment, which can be visualized in 2D (see Figure 4) and 3D (see Figure 5) graphical representation.

### 4.1. Data

BAT provides the following data:

- Time: the time in seconds since the start of the mission.
- Yaw, Pitch, Roll: BAT’s orientation in degrees around the global axes *y*, *x*, and *z*.
- $V_{x}$, $V_{y}$, $V_{z}$: the velocity relative to the global coordinate system, in m/s.
- Ranges: the range (distance) in meters from the closest object in six directions (up, down, left, right, front, and back) with respect to BAT’s current position and orientation. We use $R_{front}$ to denote the range to the object in front of BAT, similarly for all the other directions.

### 4.2. Geometrical Model

The data provided by BAT requires processing to create a geometric model. The geometric model contains the position of the drone at each timestamp, relative to its starting point, and all the range-measurements sensed by BAT. To determine BAT’s position in the *x* axis, $P_{x}$, the following formula is used:

(2) $$\begin{array}{c} P_{x}\left[i+1\right]=P_{x}\left[i\right]+\Delta t_{i}{\dot{V}}_{x}\left[i+1\right] \end{array}$$

where brackets denote the timestamp index, and

(3) $$\begin{array}{c} \Delta t_{i}=Time\left[i+1\right]-Time\left[i\right] \end{array}$$

$P_{y}$ and $P_{z}$ are computed using the same method. In order to add the objects that were in BAT’s proximity to the geometrical model, each provided range is projected from BAT’s position and orientation to the global coordinates using the following method. First, the ranges are converted to a vector representation, in $R^{3}$, in relation to BAT’s position and orientation, such that the front range is converted to $\vec{R_{front}}$: $\left[\begin{array}{c} 0\\ 0\\ R_{front} \end{array}\right]$. Similarly, all other ranges are converted to a vector representation with respect to their direction. That is, ranges in opposite directions (e.g., front and back) have opposite signs, and ranges of different axes are transformed to vector representations with non-zero values at different entries (e.g., $\vec{R_{down}}$: $\left[\begin{array}{c} 0\\ -R_{down}\\ 0 \end{array}\right]$). The resulting vector $\vec{R}$ is transformed by

$$\left[\begin{array}{c} O_{x}\\ O_{y}\\ O_{z} \end{array}\right]=\vec{R}\left[\begin{array}{ccc} \cos\alpha \cos\beta & \cos\alpha \sin\beta \sin\gamma -\sin\alpha \cos\gamma & \cos\alpha \sin\beta \cos\gamma +\sin\alpha \sin\gamma \\ \sin\alpha \cos\beta & \sin\alpha \sin\beta \sin\gamma +\cos\alpha \cos\gamma & \sin\alpha \sin\beta \cos\gamma -\cos\alpha \sin\gamma \\ -\sin\beta & \cos\beta \sin\gamma & \cos\beta \cos\gamma \end{array}\right]+\left[\begin{array}{c} P_{x}\\ P_{y}\\ P_{z} \end{array}\right]$$

After the calculation is performed, the geometrical model is used for composing visualizations of BAT’s environment.

### 4.3. 2D

The 2D visualization is a top-down view of the environment (see Figure 4 for an example). This visualization is easier to understand, but it does not represent the objects’ and obstacles’ heights. Once the mapping station receives BAT’s map, an offline loop-closure algorithm (see [25,26]), can be used to improve the map and reduce the drifts—on the mapping station side.

### 4.4. 3D

The 3D visualization is a perspective view of the environment. This visualization provides a more complete and realistic view of the environment, but it might be more difficult to perceive the building scheme as a change in the *Y*-axis (i.e., going up or down) results in the walls visualized at different heights. In addition, it is hard to differentiate between a real object and noise caused by inaccurate data.

### 4.5. Expected Map Accuracy

In this subsection, we elaborate on the expected accuracy of the mapping as performed by BAT. As mentioned, the coordinate system of the map is defined by the starting point (and orientation) of BAT. The map is constructed using several sensors, with the following expected error and drift properties:

- IMU: in particular the gyro measuring the yaw has an expected drift of about 1${}^{\circ }$/min. Note: the pitch and the roll values are not drifting as they are measured with respect to the earth gravity with an expected accuracy better than half a degree. Due to the small size of our BAT and the use of brushed motors, the use of magnetic field sensors are unreliable and therefore are not in use in most micro-drones.
- Optical Flow: has a drift which is correlated to the light and the ground texture conditions. In most cases the error is below 10% of the distance.
- Barometer: evaluating the relative height from the air pressure as measured by the barometer may result with a drift of up to 10 cm a minute, yet from our tests during a 10 min flight the expected attitude error is usually smaller than 30 cm.

The output map, as computed by the drone after flying for approximately 5 min in an average speed of 0.5 m/s, allows us, in most cases, to compute a map with an expected error of 1–2 m.

## 5. Experimental Results

In this section, we present two sets of experiments, one in simulation (using a simulated BAT) and the other is a field test of BAT in the real-world.

### 5.1. Simulation

Using the Microsoft AirSim platform, we developed a custom indoor 3D map. We further developed a model that simulates all the features of the real-world BAT, including the range-deck and the Tello drone, using the true physical parameters, such as the drone mass and dimensions. The simulation includes the following components:

- A modeling of the indoor environment, which includes obstacles and a starting point.
- BAT’s state, which includes its position, velocity and orientation, as well as the sensor reading, with artificially added noise.
- BAT’s autonomous flight-controlling algorithm.

The developed simulator allows us to first test the algorithm in the simulated environment, and only if the performance in the simulator is satisfying, to deploy the same algorithm on the real BAT. This method eliminated hardware problems, and allowed us to solve the algorithmic part first, and only then to deal with the real-world hardware-related issues.

**Figure 6:**
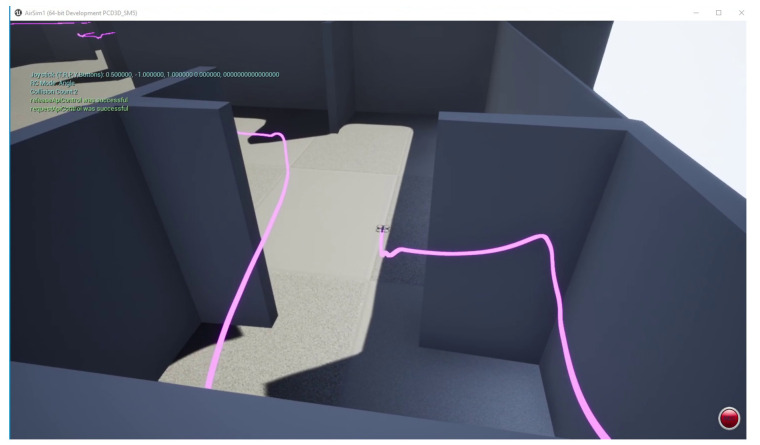
A 3D screenshot of the simulation. The magenta line represents the path of the simulated BAT.

Figure 6 presents a screenshot of the simulation. The simulated BAT successfully flew in the simulator without colliding with obstacles, and managed to explore a complex building with an area of 600 m${}^{2}$ over a period of 5 min, see in [27] for the complete BAT simulation.

### 5.2. Real-World

**Figure 7:**
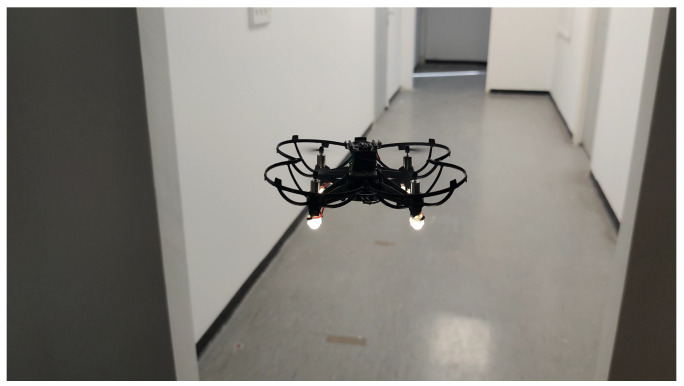
BAT exploring a corridor.

**Figure 8:**
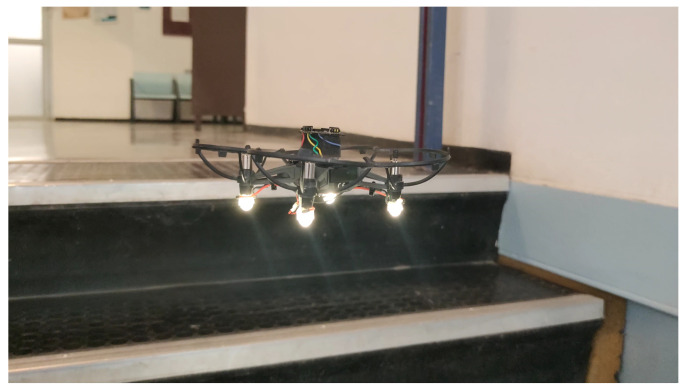
BAT flying up stairs.

**Figure 9:**
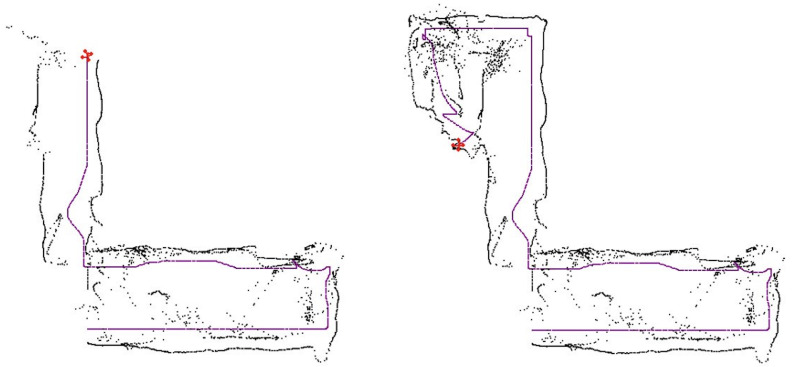
A 2D mapping by BAT, performed on a two story building: BAT’s position is marked in magenta; the left and right sensor-ranging are marked in black. **Left**: BAT has mapped the right side of the first floor and then turned right and started to go up-stairs; **Right**: BAT reached the second floor. Note that there are several “noisy spots" due to students following BAT.

**Figure 10:**
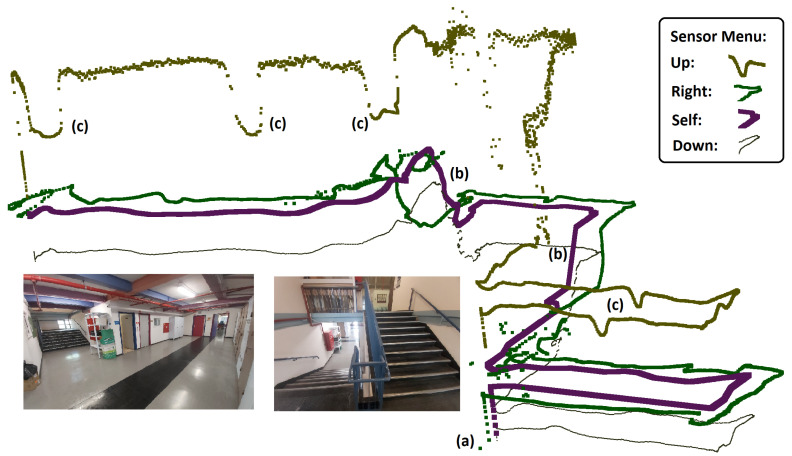
A 3D mapping of a two story building. BAT’s up, right, down and self position sensor-ranging are shown in brown, green, black and magenta lines, respectively. BAT’s starting point is marked by (a), (b) marks the stairs to the second floor—as shown in the lower right image. Changes in the ceiling height are marked by (c).

The real-world testing was performed indoors in 7 different buildings. Recall that all the computation is performed on the microcontroller of BAT, and it did not receive any external commands (see Figure 7 and Figure 8). BAT was able to fly for approximately 5 min at a time (until the battery was drained out), without crashing and without returning to the same spot twice. See [28] for a video of BAT exploring a two-story building and flying up the stairs from the first floor to the second, without crashing into the walls. BAT successfully explored all the buildings, and its collected data was used to compose a mapping of these buildings. Figure 9 shows the 2D mapping as transmitted by BAT to the mapping-station. Figure 10 shows the 3D mapping as transmitted by BAT and presented using a point-cloud viewer. During BAT’s flight there were between three to four students watching BAT and following it; their main locations were captured by BAT.

## 6. Conclusions and Future Work

This paper presents a vision-less autonomous mapping tiny-drone named BAT: Blind Autonomous Tiny-drone. Instead of using a camera, BAT uses six LiDAR sensors for navigation as well as mapping. We show that BAT is able to achieve high-quality mapping both in simulation and a field test. Motivated by bio-inspired robotics, BAT can fly autonomously in complex regions such as buildings and tunnels.

By limiting the sensory data to 12 channels (six LIDARs, optical flow, and IMU) BAT can process the data efficiently using a low power and light-weight microcontroller. Such a companion computer allows BAT to perform relatively complicated tasks such as navigating, mapping, and exploring unknown regions.

This paper raises a set of interesting future work. Our current work involves developing a Graph-Slam [29] on the sparse LiDAR data in order to allow the improved BAT to learn and recognize known places and navigate accordingly.

From an engineering point of view, it would be interesting to scale down the suggested BAT as much as possible in order to increase its maneuverability and crash resistance. On the other hand, replacing the LiDARs by a ToF matrix ranging sensor such as Pico Flexx [30], should allow the improved BAT to perform a real 3D reconstruction of the environment.

A natural generalization of this work includes using several BATs in the same region. This can accelerate the mapping process (assuming the relative initiate position and orientation of each BAT is known). Similarly to the concept of using several vacuum cleaning robots in the same building, performing a cooperation between drones in the same region and sharing data among them is the current challenge we are working on.Another interesting direction is to improve the presented framework by using reinforcement learning. Reinforcement learning is a machine learning based method that can be applied to dynamic environments, and allows the algorithm to improve its performance by interacting with the environment [31]. Deep reinforcement learning-based methods have recently gathered great success in several domains [32,33,34,35], and they are extremely useful when there is a complex objective function such as in training autonomous vehicles [36] and drone control [37]. We believe that such an approach will make the system more robust to changes.

## Figures and Tables

**Figure A1:**
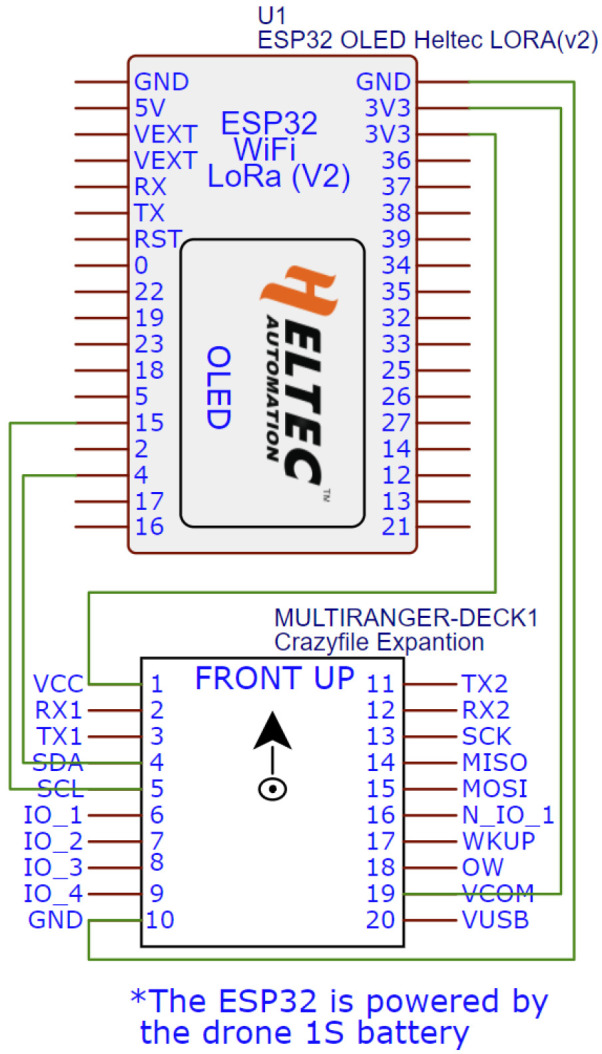
Wiring Diagram. The sensor has 5 pins: PWR, GND, SDA, SCL, and XSHUT. The XSHUT pin can be used to hold the sensor in standby mode, useful for setting up multiple sensors.

## Appendix A

### Appendix A.1. LiDAR Sensor

The VL53L1X sensor contains a sensing array of SPADs (single-photon avalanche diode), an integrated 940 nm invisible light source based on an eye-safe Class 1 VCSEL (vertical cavity surface-emitting laser) and a low-power embedded microcontroller. For every measurement, the laser beam is emitted from the VCSEL and reflected back from an obstacle to the 16 × 16 SPAD array, the embedded microcontroller derives the distance and accuracy of the measurement and sends it to the HELTEC microcontroller (on the $I^{2}C$ bus).

The sensors are configured in a continuous ranging mode, an “inter-measurement period” and “ranging duration” (timing budget) can be set. Optimizing each of these parameters can help with measurement accuracy and repeatability, for example, on the one hand a longer inter-measurement period allows ranging to a farther distance, but on the other hand, this may result in a reduction of measurement frequency making it less reliable to fly smoothly.

All of the vl53l1x sensors have the same default $I^{2}C$ address (0x29), running all the sensors simultaneously will raise an address conflict, therefore, every sensor must be assigned a unique $I^{2}C$ address. To overcome this issue, the on-board pca9534 $I^{2}C$ GPIO Expander is utilized, as all the vl53l1x sensors XSHUT pins are connected to pca9534 I/O pins and can be controlled by software. On startup the sensors are cycled one by one, released from standby mode and assigned a new $I^{2}C$ address along with the sensor setup.

### Appendix A.2. Wiring

The ESP32 is powered by the drone’s 1s battery, a pair of wires were soldered to the positive and negative battery connector on the drone PCB. The multi-ranger deck $I^{2}C$ SDA and SCL are connected to pins 4 and 15 respectively, as well as the power pins. VCOM (on the multi-ranger deck) must also be connected to a 3.3 V power pin as it is required for properly powering the sensors. See Figure A1 for additional information.

### Appendix A.3. Algorithm Constants

#### Appendix A.3.1. PID Values

For the Pitch, we set $K_{p}$ to 15, $K_{i}$ to 0.04, $K_{d}$ to 4, and *d* to 0; for the Roll, we set $K_{p}$ to 60, $K_{i}$ to 60, $K_{d}$ to 10, and *d* to 0.5; and for the Throttle, we set $K_{p}$ to −0.5, $K_{i}$ to −0.04, $K_{d}$ to −0.1, and *d* to 1.

#### Appendix A.3.2. Control Loop Values

We set the control loop constants as follows:

- emergency_threshold:=0.3
- front_threshold:=1
- tunnel_threshold:=0.25
- right_far_threshold:=2.5.
